# Telemedicine in allergology: practical aspects: A position paper of the Association of German Allergists (AeDA)

**DOI:** 10.1007/s40629-021-00167-5

**Published:** 2021-02-22

**Authors:** Stephanie Dramburg, Uso Walter, Sven Becker, Ingrid Casper, Stefani Röseler, Astrid Schareina, Holger Wrede, Ludger Klimek

**Affiliations:** 1 Department of Pediatric Pneumology, Immunology and Intensive Care Medicine, Charité—Universitätsmedizin Berlin, Augustenburger Platz 1, 13353 Berlin, Germany; 2 Practice for Otorhinolaryngology Walter and Sachse, Duisburg, Germany; 3 Department for Otorhinolaryngology, Head and Neck Surgery, University Medical Center, Tübingen, Germany; 4 Center for Rhinology and Allergology Wiesbaden, Wiesbaden, Germany; 5 Clinic for Pneumology, Allergology, Sleep and Respiratory Medicine, Augustinians Hospital, Cologne, Germany; 6 Practice for Internal Medicine and Allergology, Cologne, Germany; 7 ENT and Allergy Center Herford, Herford, Germany

**Keywords:** Allergy care, COVID-19, Video consultation, eHealth, Digital health

## Abstract

Since spring 2020, the wide-ranging contact restriction measures in the context of the severe acute respiratory syndrome coronavirus 2 (SARS-CoV-2) pandemic have also led to a reduction in physician–patient contacts in the ambulatory care setting. Telemedicine applications will increasingly provide a way to efficiently deliver patient care under infection control measures. In allergology, telemedical as well as digital applications can also significantly facilitate everyday clinical practice. However, the technical and legal hurdles associated with the implementation of digital strategies must be overcome for this to happen. The aim of this article is to provide an intuitive overview of the aspects to be considered in the implementation of telemedicine consultations and to highlight the current state of the framework as well as optimization possibilities and perspectives in allergology. If a structured use is guaranteed, digital and telemedical applications can improve patient care—also in allergology. There is potential to be exploited in many areas, from the remote collection of clinical history, and video consultations, to the discussion of diagnostic findings, disease monitoring, and therapy support. The use of telemedical applications, especially video consultations, has experienced a remarkable acceleration in the context of the coronavirus disease 2019 (COVID-19) pandemic. The present overview of the legal, technical and professional framework is intended to support the anchoring of digital and telemedical technologies in everyday allergology. However, in order to consolidate these in the future, an agreement is needed regarding professional standards of action as well as a remuneration structure that is permanently defined beyond the current pandemic.

## Telemedicine in times of pandemic-related contact restrictions

**Fig. 1:**
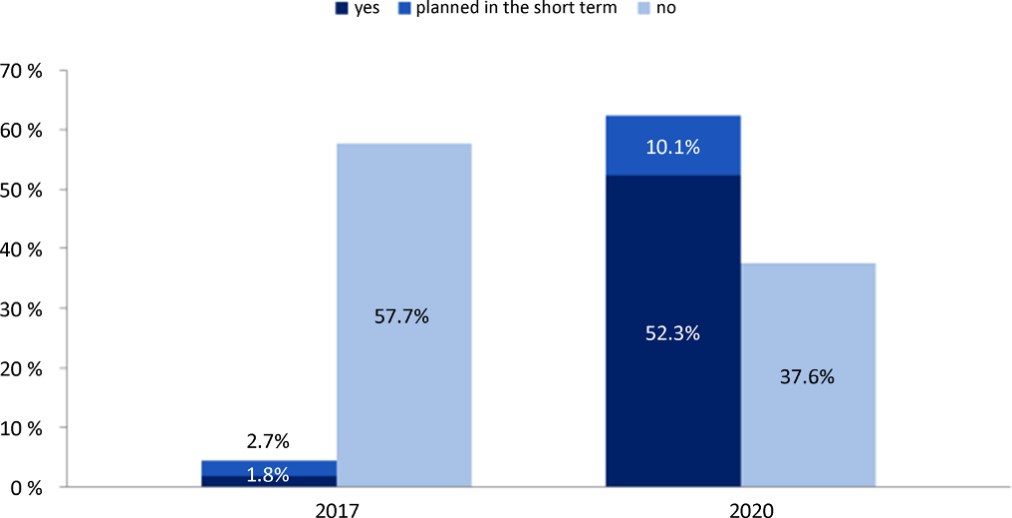
Use of video consultations—comparison of surveys from 2017 and 2020: results of representative surveys among physicians from different specialties conducted by “Stiftung Gesundheit” (German Health Foundation) (2017) in collaboration with the Health Innovation Hub of the German Federal Ministry of Health (2020). (2017 *n* = 198, 2020 *n* = 2128, from [3]. Note: In the 2017 survey, there were additional response options (e.g., “I can well imagine”) that are not shown here)

In spring 2020, the extensive contact restriction measures adopted by the German federal government and many other countries in the context of the SARS-CoV‑2 pandemic led to a reduction in COVID-19 incidence and consequently in the number of patients requiring inpatient treatment. At the same time, however, the pandemic and associated societal changes also had a marked impact on outpatient care. In particular, in the German ambulatory–elective setting, the number of physician–patient contacts decreased rapidly [1], while emergency physicians observed with concern how the caution of the population also manifested itself in decreasing case numbers in emergency departments [2]. Underuse of medical care in the area of noninfectious, chronic, and acute conditions was looming. In order to maintain sufficient, comprehensive patient care in the outpatient setting, even under infection control measures, interest in telemedicine applications increased to a remarkable extent compared with previous years (Fig. 1; [3]). This development was supported by a rapidly growing range of certified technology providers as well as corresponding remuneration incentives and simplifications in the organizational process with the Associations of German Statutory Health Insurance Physicians.

An essential prerequisite for the use of telemedicine in Germany was the decision to loosen the ban on exclusively remote treatment at the 121st German Medical Congress [4], even before the start of the SARS-CoV‑2 pandemic. The concrete implementation of the resolution was subsequently carried out by the state medical associations. In 2019, the Digital Healthcare Act came into force with the aim of advancing digitization in healthcare [5]. The focus here is set on telematics and telemedicine, with the declared aim of strengthening intersectoral collaboration and the provision of care in structurally weak areas. In the light of the SARS-CoV‑2 pandemic, particularly the advantage of contact-reduced treatment came to the fore.

Current surveys show a good acceptance of telemedical applications in different areas of healthcare and across different professions related to patient care [6]. It is however clear that the used technologies differ depending on the user group and clinical scenario [7]. In addition to communication and networking services, which are particularly used at the interface between inpatient and outpatient care, video consultation is probably the most important use of telemedicine in ambulatory settings.

The aim of this guide is to present the technical and legal prerequisites for successful patient care using video consultations and to explain these on the basis of practical examples in order to support first experiences in telemedicine.

## Definition and forms of telemedicine

Since telemedicine brings together a wide range of different concepts and methods of care, there is always a lack of clarity in the concrete definition of the term, especially when it comes to distinguishing it from other areas such as “mobile health” (mHealth) or “electronic health” (eHealth). In order to counteract confusion, the Telemedicine Working Group of the German Medical Association defined telemedicine in 2015 as a “… collective term for various medical care concepts that have in common the basic approach of providing medical services for the population in the areas of diagnostics, therapy and rehabilitation, as well as in medical decision-making advice over spatial distances (or temporal offset). Information and communication technologies are used in this process” [8].

**Fig. 2:**
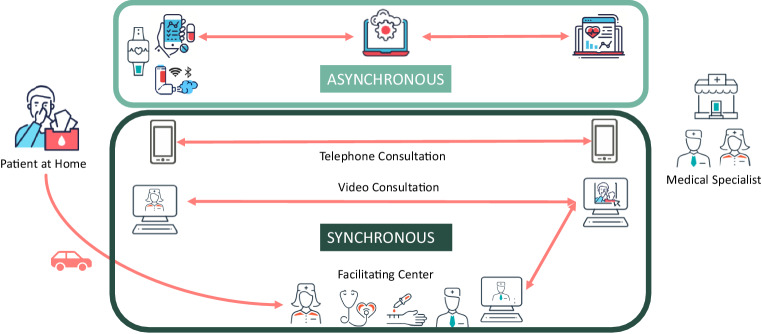
Forms of telemedical applications based on [21]

Telemedicine concepts can generally be divided into three areas: asynchronous, synchronous and facilitated synchronous (Fig. 2). The most commonly used applications in the field of synchronous telemedicine are telephone consultations and video consultations. A supplementary method, which is used particularly in the USA due to greater distances to specialized care centers, is “facilitated telemedicine”. Here, physical examinations and diagnostic tests (e.g., lung function tests, skin prick test, blood sampling, etc.) are performed by a healthcare professional on site and the results are discussed with a corresponding specialist via video consultation. However, this approach has not yet been widely used in Germany.

Another variant of remote consultation is the asynchronous answering of medical questions, for example via chat or e‑mail. Here, there is no direct doctor–patient contact, but instead questions from the doctor are answered via apps with a time delay and monitoring data or findings are uploaded to cloud servers. This offers increased flexibility on the part of the medical staff, but can only be used for very selected questions and is associated with a significantly limited ability to assess the clinical findings. Asynchronous methods are not suitable for replacing the doctor’s visit, but can be useful for (re)processing specific topics in known patients.

The efficacy of telemedicine care compared to face-to-face care has been demonstrated in asthma management [9] and clarification of suspected penicillin allergy [10, 11]. Promising results were also shown in the follow-up of COVID-19 patients [12] and in dermatological care [13].

## Suitable settings for video consultations

The careful selection of suitable scenarios and patients is essential for the responsible and smooth use of telemedical procedures [14]. In principle, only patients who do not need to undergo a physical examination should be scheduled for a video consultation (especially during the initial visit, but also during follow-up visits if these require a new physical examination). The remote consultation is thus ideally suited, for the following settings: (i) discussion of diagnostic test results which arrive from other healthcare providers or with a certain delay after obtaining the specimen (e.g., laboratory results, imaging, etc.), (ii) evaluation of therapy success and (iii) adherence to longer-term therapies before further prescriptions of medications (e.g., sublingual immunotherapy, SLIT; biologics therapy) and (iv) general counseling of patients. With regard to the possibility of a general consultation via video, the clinical picture as well as the current manifestation or symptom control is decisive. In case of a suspected acute reaction and anaphylaxis in the history, as well as in case of an asthma exacerbation or severe symptom expression of other allergic diseases, the personal presentation is preferable. In order to make the clinical workflow in the clinic or practice economical, video consultations should be offered in blocks, for example, morning attendance consultation, afternoon video consultation. Delays caused by a “media disruption” can thus be minimized.

In addition to deciding on the clinical suitability of patients, some general and technical aspects must also be considered. For example, the individual possibilities and abilities of the patient: Is an appropriate medium (e.g., cell phone with camera, PC with camera and microphone/speaker) available for a video consultation? Is the patient able to connect online and operate the device? Does the patient want to be attended by video or prefer face-to-face contact? An overview of suitable scenarios for a mix of face-to-face contact and video consultation can be found in Table 1.

**Table 1:** Selection of medical actions in allergology and their possible distribution of medical actions in the practice or video consultation hour

Clinical setting	At the clinic/private practice	During a video consultation
First consultation by the allergist	Diagnostic tests	Clinical history
		Consultation regarding diagnostic tests
(Dietary) advice in the case of known food allergies	Diagnostic tests (especially in case of unknown trigger or first reaction)	Nutritional advice
	Treatment of acute systemic reactions	Clarification of individual questions (e.g., the introduction of new foods in childhood)
	Discussion and adaptation of the emergency plan	
Follow-up of atopic dermatitis	Diagnostic tests	Control of the skin’s appearance
	Initial physical examination	Orientational care advice
	Follow-up for severe cases	Therapy adaptation
	Evaluation of patients for whom a visual evaluation via video is not reliably possible	
Follow-up of allergic asthma	Diagnostic tests	Review of progress
	Acute exacerbation beyond the control of the asthma action plan	Discussion of findings
	Poorly controlled asthma (ACT < 20)	Review of inhalation technique and peak flow measurements
	Acute PEF-reduction (>20%)	
SLIT	Diagnostic tests	Therapy and adherence monitoring
	Initiation of therapy	Issuing of new prescriptions (shipping by post or e‑prescription in the near future)
SCIT	Diagnostic tests	Discussion of findings
	Application of injections	Therapy monitoring
Treatment with biologics	Diagnostic tests	Support for the first injections carried out independently at home
	First injection	Therapy monitoring
	Evaluation therapy success after 16 weeks and one year	Follow-up prescriptions (post/e-prescription)

*ACT* asthma control test, *PEF* peak expiratory flow, *SCIT* subcutaneous immunotherapy, *SLIT* sublingual immunotherapy

## Best practice example: treatment with biologics

**Fig. 3:**
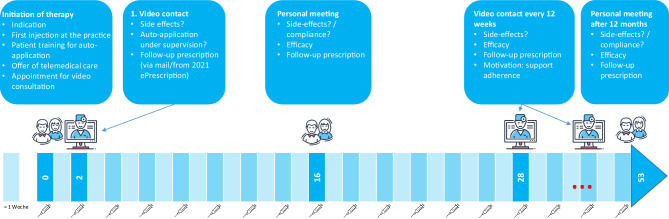
Example of a therapy scheme for biologics therapy with integrated video consultation

In the context of a therapy with biologics, video consultations can be used excellently to reduce direct patient contacts to the necessary extent, to improve adherence and to document the therapy progress. The following scheme can be used for a therapy with biologics (Fig. 3):

After the indication has been established, the first dose is administered in the practice by the patient after prior instruction by the physician. The patient is informed about the possibility of follow-up via video consultation and about the technical and data protection aspects. If the patient consents to a telemedical consultation, he or she signs the required consent and data protection forms. At the end of the presentation, the first video consultation is scheduled in 14 days.

At the agreed date, the tolerability is checked during a video consultation. In addition, there is the possibility of online monitoring of the next application by the patient in the telemedical presence of the physician. Further applications are then carried out by the patient himself.

After 16 weeks, the success of the therapy and possible side effects are reviewed in a personal meeting in the practice. If the patient is satisfied with the telemedical care, the next video appointment is arranged at the appropriate interval (for example, for therapy with dupilumab after further six injections—12 weeks). In this case, another prescription is sent by mail.

A face-to-face re-visit to the practice is targeted after 6–12 months, depending on symptom progression. In such an alternation of face-to-face and telemedical follow-up appointments, the treating physician retains control over the therapy at all times, while the patient benefits from low-threshold access to medical advice.

## Best practice example: sublingual immunotherapy

**Fig. 4:**
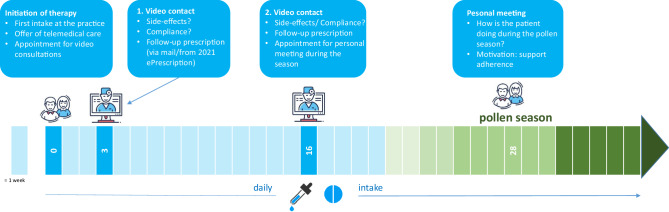
Therapy scheme of a sublingual immunotherapy against early bloomers with integrated video consultation

Also in the context of sublingual immunotherapy (SLIT), video consultations can be used very well to reduce contact while maintaining therapy and adherence control. In addition, corresponding smartphone applications for recording symptoms and medication intake (e.g., ‘Husteblume’ or ‘MASK-air’, no claim to completeness) can be used, which provide both the patient and the treating physician with an objective overview of the observation/adherence period. Using the concrete example of SLIT for birch and other allergens with a similar flowering period, the following scheme has proven effective (Fig. 4):

After establishing the indication (usually in autumn), the first dose is administered in the practice by the beginning of February at the latest. Within the framework of this appointment, the offer of a telemedical follow-up takes place and in case of acceptance, a timepoint for the first remote follow-up is agreed.

Tolerance and adherence are checked after 3 weeks during a video consultation. If the doctor and patient are satisfied with the care over a distance, the tolerance and adherence are checked again after 16 weeks via video call and the follow-up prescription is sent by mail. In addition, a personal presentation will be scheduled in the practice during the pollen season. During the personal presentation, further (symptomatic) medication can be prescribed, if necessary, and further follow-up can be discussed or scheduled via video.

## Technical aspects of a video consultation

A video consultation should largely correspond to the consultation situation in the practice. In addition, basic technical requirements must be met (Table 2).

**Table 2:** Checklist of technical requirements for the successful implementation of a video consultation

Certified technology provider
Calm and private room
Good light and sound quality
Stable internet connection
*Further information (in German)*
Information from the National Association of Statutory Health Insurance Physicians on video consultation: https://www.kbv.de/html/videosprechstunde.php
List of certified technology providers: https://www.kbv.de/media/sp/Liste_zertifizierte-Videodienstanbieter.pdf

### Suitable premises

The room must provide sufficient privacy. There should be as little disturbance as possible from outside noise, phone calls or other people’s activities. The workstation within the room should be well lit, ideally from behind the screen, and provide a neutral background. If possible, it should be possible to access patient data during the video consultation. This can be ensured, for example, by a second screen.

### Suitable hardware

Video consultations can be performed on a PC or laptop with the best possible camera, integrated microphone and loudspeaker. To ensure good image quality, it may be advisable to purchase an additional camera, depending on the computer’s equipment. To ensure good sound quality and avoid reverberation effects, it is recommended to use headphones with an integrated microphone (headset). This is particularly useful when talking to elderly and hearing-impaired patients.

### Stable internet connection

As a general rule, LAN (cable-based) is better than WLAN (wireless). Since images and sound are transmitted, a sufficiently powerful Internet connection must be provided. Unless necessary, other programs programs with a connection to the Internet should be closed during the video consultation (telecommunication services, Dropbox, etc.).

### Certified software

When selecting the software, make sure that it is certified. In Germany, for example, the National Association of Statutory Health Insurance Physicians provides a regularly updated list of providers on its website. Given the abundance of offers, it is important to consider in advance, which functions will be needed, as this also determines the price.

Possible functions in addition to the basic teleconference tools are the following: (i) group conferencing, (ii) screen sharing to present findings to the patient, (iii) chat functions to provide written information or share links, and (iv) automated appointment scheduling.

With many providers, making individual calls is free of charge. Otherwise, there are both subscription models and individual call charges. The selection of the most cost-efficient solution depends above all on the frequency of use and the range of functions offered by the software. If these points are all fulfilled, nothing stands in the way of an undisturbed doctor–patient communication.

## Procedure of a video consultation (doctor–patient)

Telemedical doctor–patient contacts should be prepared and carried out professionally. This includes functioning technology in a professional surrounding. Physicians and patients should be aware that virtual interaction has its opportunities and limitations. It is therefore important to clearly define the process of a telemedical contact between patient and physician.

This begins with planning: follow-up contacts are ideally suited, while the initial contact between physician and patient should ideally take place in the context of a face-to-face appointment, as it usually includes a physical examination and, if necessary, further diagnostics. However, if the first visit is followed by a discussion of the findings and the determination of further diagnostics or therapy planning (e.g., allergen-specific immunotherapy), a telemedical contact is a suitable option.

The offer for this should be made by the treating party through the physician, via the medical staff or practice information systems (waiting room TV, brochures). Since December 2019, the advertisement of remote treatment is permitted in Germany, for example on the practice website, if, according to generally accepted professional standards, personal medical contact with the person to be treated is not necessary and, according to the accepted state of medical knowledge, proper treatment and consultation using communication media is fundamentally possible [15].

If the patient agrees to a remote contact, he or she should ideally sign or submit a corresponding consent and data protection declaration during the initial contact in the practice, but at the latest before the video consultation begins. Many certified technology service providers already offer standard forms integrated into the software for this purpose. Consent to remote treatment can also be given verbally, but should be documented in the patient file. Appointments for a virtual consultation are made online or via the medical assistants, depending on the technology service provider and the preference of the practice. For patients without prior face-to-face contact, a brief telephone assessment should first be made to determine whether the patient’s condition and suspected clinical picture allow adequate care via video contact or whether a face-to-face appointment is preferable. Generally, this decision is the responsibility of the physician, who can, however, delegate the assessment under adequate guidance (for the more detailed technical procedure, see below.).

When opening the virtual consultation, after welcoming the participants, it is advisable to clearly describe the topic to be discussed and also, to mention the scheduled time frame. The physician should be aware of the limited nonverbal communication in the digital exchange with the patient. Primary nonverbal communication takes place online via the voice and facial expressions, to which the technology should be adapted accordingly. Care should be taken to design the room lighting accordingly to enable good visibility of facial expressions. Light sources located behind the computer are suitable for this purpose. Furthermore, a discussion guide/record is suitable, which is kept by both participants. The patient may be able to download this online from the practice website in advance, or it may be given to him in the practice. This allows the patient to be aware of their questions in advance and actively prepare for the online appointment. The questions that need to be clarified, for example, findings of the laboratory tests, are named and discussed between the patient and the doctor. Finally, a summary of the topics discussed is recommended, as well as an outlook on the next treatment steps and whether these should again take place via telemedicine or in person.

## Procedure of a video consultation (doctor–doctor)

Like the above-mentioned telemedical contacts between doctor and patient, telemedical exchanges between medical colleagues should also be clearly stated in terms of their advantages and limitations and carried out in an appropriately professional manner.

The goal is direct contact between physicians without being limited by spatial and temporal boundaries. The targeted, professional exchange is intended to enable more effective cooperation between various healthcare structures (clinic, practice, specialized outpatient clinic, etc.) and the physicians involved.

As with patient–doctor contact, the procedure here should also be effective and time-saving. An appointment should be made in advance, naming the patients and/or topics to be discussed. It should also be clear which persons will participate in the online meeting. The time and framework should be clearly stated and adhered to. Waiting times should be avoided, especially online. Any findings, images, media to be demonstrated should be ready and presentable. A protocol or checklist during the telemedical consultation can be helpful. Once the questions to be clarified have been discussed, the end of the telemedical consultation should also be actively initiated, possibly by means of a previously defined time frame.

The aim of direct contact in telemedicine—for example, between allergists in private practice and senior physicians in a specialist outpatient clinic—is to enable an exchange at eye level. The lower the inhibition threshold for telemedical contact is set, the more likely it is to succeed in introducing even traditionally “analog” colleagues to the possibilities of this modern form of communication.

## Legal framework

Exclusive remote treatment has been permitted under professional law in Germany since May 2018 and has been integrated accordingly into the professional regulations of the state medical associations [16]. The state of Brandenburg is an exception to this. However, it should be mentioned, that the use of telemedical applications for known patients is also allowed in Brandenburg, as the probition only applies to exclusive remote treatment.

In § 7 (4) sentence 2 of the Model Professional Regulations for Doctors, it is emphasized that personal doctor–patient contact remains the gold standard, which is to be supported but not replaced by the use of digital technology. Thus, permission for exclusive remote treatment is an exception to the general regulation, which applies subject to the following conditions: the exclusive remote treatment of previously unknown patients must (i) be medically justifiable, (ii) medical diligence must be maintained, and (iii) patients must be fully informed about the special features of this type of treatment.

### Privacy

The protection of sensitive, personal health data is essential in everyday clinical practice. In concrete terms, this means that only certified communication technologies should be used that comply with the requirements of the General Data Protection Regulation (GDPR, Europe-wide) and the German Federal Data Protection Act (national implementation). With regard to telemedical services, for example software providers for video consultations, physicians should make sure to choose a provider listed by the German Association of Statutory Health Insurance Physicians. However, care should also be taken when communicating within the practice team and external colleagues. Although quick access to popular short messaging services such as WhatsApp is not ruled out under data protection law if the patient has been informed accordingly and has consented to the use of this form of communication, conflicts arise here under professional law because the storage and possible use of the data by third parties cannot be explained transparently. Thus, the user may be in breach of confidentiality, as access to sensitive health data by third parties is possible. Particular attention must also be paid to the correct encryption of e‑mail content if this is used, for example, to send medical reports. Some state medical associations offer good advice on this, and the practice check of the German Medical Association can also be consulted [17].

As soon as a medical center employs more than 20 people who regularly handle patient data, a data protection officer should be appointed. This can be an external service provider, or an employee of the practice can perform the function after appropriate qualification. The task of the data protection officer is to regularly check compliance with the GDPR in everyday practice and to discuss any new issues that arise [18].

### Liability

The requirements for telemedical consultation and/or treatment correspond to the previous concept of care for analog treatment. In accordance with the treatment contract, § 630a ff. BGB, the physician is thus obligated to comply with the generally accepted professional standard (medical indication/patient consent/adherence to the specialist standard) during treatment. In addition to the duty of care, the duties to inform and clarify, § 630c, 630e BGB, also apply in accordance with treatment based on personal contact.

In addition, there are further duties of care in connection with telemedical remote treatment. In particular, these relate to the duty to obtain findings, documentation and information. In summary, the attending physician is obligated to decide whether remote treatment is justifiable for the purpose of ascertaining findings. In addition, the patient must be informed about the benefits, but also the limitations and risks of remote treatment, and the medical documentation is expanded to include these aspects. Further information on this can be found in the online material of the Association of Statutory Health Insurance Physicians and at [19].

Since no generally applicable, professional, medical standard has yet been defined for telemedicine, the due diligence requirements developed by case law for new treatment methods apply. These oblige the practitioner to exercise the care of a prudent practitioner—specified to the circumstances of the telemedical treatment method. In the event of a breach of the duty of care, the physician is liable in contract and, in principle, also in tort [19].

### Remuneration and billing

In principle, video consultations are billed in the same way as services provided in the practice (Table 3), in accordance with the German Standard Scale of Fees (EBM) and the German Fee Schedule for Physicians (GOÄ). However, by no means can all services be provided by telemedicine. Since the increased use of video consultation hours is politically desired, additional fee schedule items (GOP) have been created. These consist of a technology surcharge, starting support and an administrative surcharge for the creation of a patient file for patients who have not yet been to the practice in the same quarter.

**Table 3:** Overview of remuneration for telemedical services in Germany

*Remuneration by Statutory Health Insurances*	
Basic rates with surcharges	Each specialist group can bill its specific basic rate if it is an initial contact
	If further treatment takes place exclusively via video consultation, the pseudo cipher 88220 must be used and the basic rate is then reduced by 20–30% depending on the specialist group
	In addition, surcharges can be charged for specialists medical care (PFG surcharge), for the performance of basic medical care and for treatment by conservative ophthalmologists. These are usually automatically rewarded by the National Association of Statutory Health Insurance Physicians
Technology surcharges, additional starting support	01444 (10 points): surcharge for the authentication of a patient who has not been seen in person during the current quarter. (limited until 01 September 2021)
	01450 (40 points): technology surcharge (max. 1899 points per quarter)
	01451 (92 points): starting support (in the case of max. 50 video consultations). The prerequisite is that at least 15 video consultations are carried out per quarter
Additional services	Consultation services such as allergological anamnesis (GOP 30100) or basic psychosomatic care (GOP 35100 and 35110)
	Psychotherapeutic services
	Case conferences such as interdisciplinary tumor or MRSA conference
*Services According to the German Scale of Medical Fees*	
Consultation	Ciphers 1, 3, 34
Organ-related examinations	Cipher 5
If necessary, reports and certificates	Ciphers 70, 75
*Additional Material*	
Overview of the National Association of Statutory Health Insurance Physicians on the remuneration of video consultations:	https://www.kbv.de/media/sp/Videosprechstunde__uebersicht_Verguetung.pdf

*GKV* statutory health insurance, *GOP* fee schedule item, *GÖA* fee schedule for physicians, *PFG* flat rate for primary care by specialists, *MRSA* methicillin-resistant Staphylococcus aureus

There are some limits on the number of GOP that can be reimbursed as part of a video consultation. Some of these limits are temporarily suspended due to the COVID-19 pandemic. In addition, the number of video-only treatment cases is limited to 20% of all treatment cases. The German Fee Schedule for Physicians (GOÄ) does not include any surcharges for video consultation hours, and an increased incremental rate is generally not accepted either.

Basically, three groups of services can be distinguished for patients with statutory health insurance (Table 3).

## Digital health applications

On December 19, 2019, the Digital Healthcare Act (DVG) came into force. This created the possibility for digital health applications (DiGA—Digitale Gesundheitsanwendung) to be included in standard care (“app on prescription”). In October 2020, the first two applications for behavioral treatment of tinnitus (Kalmeda) and digital support for anxiety disorders (Velibra), were added to the DiGA directory.

In principle, all digital medical devices of Class I and IIa meeting the following requirements in accordance with § 33a of the German Social Code, Book V can be approved and prescribed as a reimbursable healthcare app: (i) they serve to detect and treat illnesses, injuries or disabilities or support patients on their way to a self-determined, health-promoting lifestyle; (ii) the main function is based on digital technologies and (iii) the medical purpose is essentially achieved through digital functions. A prerequisite for approval is prior testing by the Federal Institute for Drugs and Medical Devices (BfArM), which is currently possible in a “fast track” procedure [20]. In the course of this review, the required product characteristics are checked on the basis of the manufacturer’s specifications (data security, usability, etc.) and the proof of effectiveness. The latter can consist of a medical benefit or a patient-relevant structure or process improvement.

If such a positive effect has already been proven, the app will immediately be included in the DiGA directory. If the proof is still pending and the positive supply effect is only plausible, the application is provisionally included in the directory. The positive supply effect must then be proven within one year by suitable scientific studies. Once the application is listed in the directory, it can be prescribed by physicians and psychotherapists on a budget-neutral basis and used by patients with statutory health insurance free of costs. In the first year, the price specified by the manufacturer is reimbursed, after which a price is negotiated between the manufacturer and the National Association of Health Insurance Funds.

The prescription of a digital health application is carried out analogously to the drug prescription by means of form 16 (“red prescription”). The listed health applications are part of the drug list of common practice management systems and can be prescribed without co-payment from the patient by stating the name, the pharmaceutical central number and the duration of use.

## Educational videos

Digitization in medicine can also be used to educate patients about diseases, treatments and medical contexts. This saves valuable medical working time, and patients can access the information at any time and from any location. At the same time, the flood of unverified medical content on the Internet can be countered by scientifically verified facts.

This form of medical education is best achieved by means of videos, since on the one hand they meet the media consumption behavior of patients and on the other hand they are easy to produce and distribute. Sophisticated videos can be produced with modern smartphones, an additional microphone and good lighting. Editing software is preinstalled on most smartphones today or can be downloaded in the form of free programs. Common commercial software like Microsoft PowerPoint can also be used to produce videos, e.g., of lectures. The videos can then be distributed via common platforms such as YouTube, Vimeo or Facebook. To do this, a (free) account or channel must be opened. It is also possible to integrate videos an own website. In order to reach the largest possible audience or to be found by interested patients, appealing titles and still images (so-called thumbnails) as well as meaningful keywords should be used (Table 4).

**Table 4:** Checklist for the successful production and publication of educational videos

Checklist: Educational Videos	
Recording	Smartphone camera with additional microphone and possibly lighting
Content	Narrative with a quick introduction to the topic and an entertaining performance
Editing	Software on the smartphone, Microsoft PowerPoint or a commercial editing software
Dissemination	Via Youtube, Vimeo, other social media platforms or the own website

In terms of content, the videos should follow a flow of information mentioning the topic in the first few seconds and, for example, causing curiosity with a provocative statement or a question and then dealing with the topic in a lively manner. Good storytelling is rewarded by viewers who remain watching more patiently. This is not unimportant, as the algorithms of Internet platforms prefer to display videos that are clicked on as often as possible and viewed for as long as possible. However, successful videos benefit not only the patients, but also the producers. This is because their videos draw attention to themselves and their medical expertise and increase their profile in the increasingly competitive medical market.

## Perspectives

Telemedicine applications are often seen as synonymous with conducting video consultations. However, the possibilities of telemedicine are much broader and will become increasingly important in the future. In addition to the advantages for medical care, there are also economic aspects that will contribute to this. After all, the bottleneck of medical care is personalized treatment, which will become less rather than more available in the future. Concepts of care supplementing or replacing certain medical activities and making them available to patients anywhere and at any time will therefore become established sooner or later. Various areas that will be increasingly characterized by telemedicine applications are listed below without claiming to be exhaustive:

### Education and counseling

The German healthcare system is already at the edge of its capacity due to the increase in chronic diseases. Helping people to help themselves is therefore a crucial aspect. This succeeds above all also through serious counseling. Informed patients relieve the burden on the system because they seek medical help less frequently and ideally learn to take responsibility for their own health. Telemedicine applications such as educational videos and apps are ideally suited for such counseling, countering the aimless Google search with meaningful health information. Independent certification of such digital products, as has already been introduced with the DiGA Directory and as the “Stiftung Gesundheit” (German Health Foundation) already offers for print media, would be desirable in this regard.

### Control

The time available for treating patients in practices and outpatient clinics is naturally limited. In addition, this treatment incurs high accompanying costs for staff, use of space and other costs. Video consultations conducted during or also outside of official office hours help save time and resources. Unnecessary travel costs and waiting times for patients are also eliminated.

### Diagnostics

Telemedical tools enable remote diagnosis to some extent. Whether screening for suspicious skin changes, interpreting pulse values and ECG recordings, vision and hearing tests, or serological point-of-care diagnostics: the list of possible applications is growing. In the case of corresponding illnesses, the digital, automated transmission of vital parameters and other medical data, for example, blood glucose levels or movement profile, enables rapid and targeted medical intervention.

### Therapy

Telemedicine applications can generate large amounts of data in a very short time, which is a fundamental prerequisite for personalized medicine. By collecting and analyzing large volumes of data (big data) supported by artificial intelligence (AI), it will be possible to individualize therapy regimens with ever greater precision in the future.

Digital health applications will establish themselves in the next few years as the fourth pillar of care alongside pharmacotherapy, psychotherapy and surgical medicine. With their help, it will be possible to treat patients much faster, more individually and more effectively. In particular, chronic diseases and psychovegetative complaints can already be treated in this way with a high level of evidence.

### Framework

Efficient bureaucratic processes and a solid reimbursement structure are essential to enable the smooth integration of digital and telemedical applications into everyday clinical practice even after the COVID-19 pandemic. Legal standards for action must be established, and digital health and telemedical treatment approaches must be anchored as part of the medical curriculum.

## Conclusion

The use of telemedical applications, especially video consultation, has experienced a remarkable acceleration in the context of the COVID-19 pandemic. The overview of the legal, technical and professional framework is intended to support the anchoring of digital and telemedical offerings in everyday allergology. However, in order to consolidate these in the future, an agreement is needed regarding professional standards of action as well as a permanently defined remuneration structure beyond the pandemic situation.

## Abbreviations

BfArM: German Federal Institute for Drugs and Medical Devices
COVID-19: Coronavirus disease 2019
DiGA: Digital health applications
DVG: Digital Healthcare Act
EBM: German uniform assessment standard for medical services
eHealth: Electronic health
GDPR: General Data Protection Regulation
GOÄ: German scale of medical fees
GOP: Fee schedule items
mHealth: Mobile health
SARS-CoV‑2: Severe acute respiratory syndrome coronavirus 2
SCIT: Subcutaneous immunotherapy
SLIT: Sublingual immunotherapy
